# Supplementary material for: Recurrent network dynamics shape direction selectivity in primary auditory cortex
Source: Nat Commun. 2021 Jan 12;12:314. doi: 10.1038/s41467-020-20590-6 (PMC7804939; doi:10.1038/s41467-020-20590-6)
Supplement: Supplementary file 1 — Supplementary Information [file 41467_2020_20590_MOESM1_ESM.pdf]

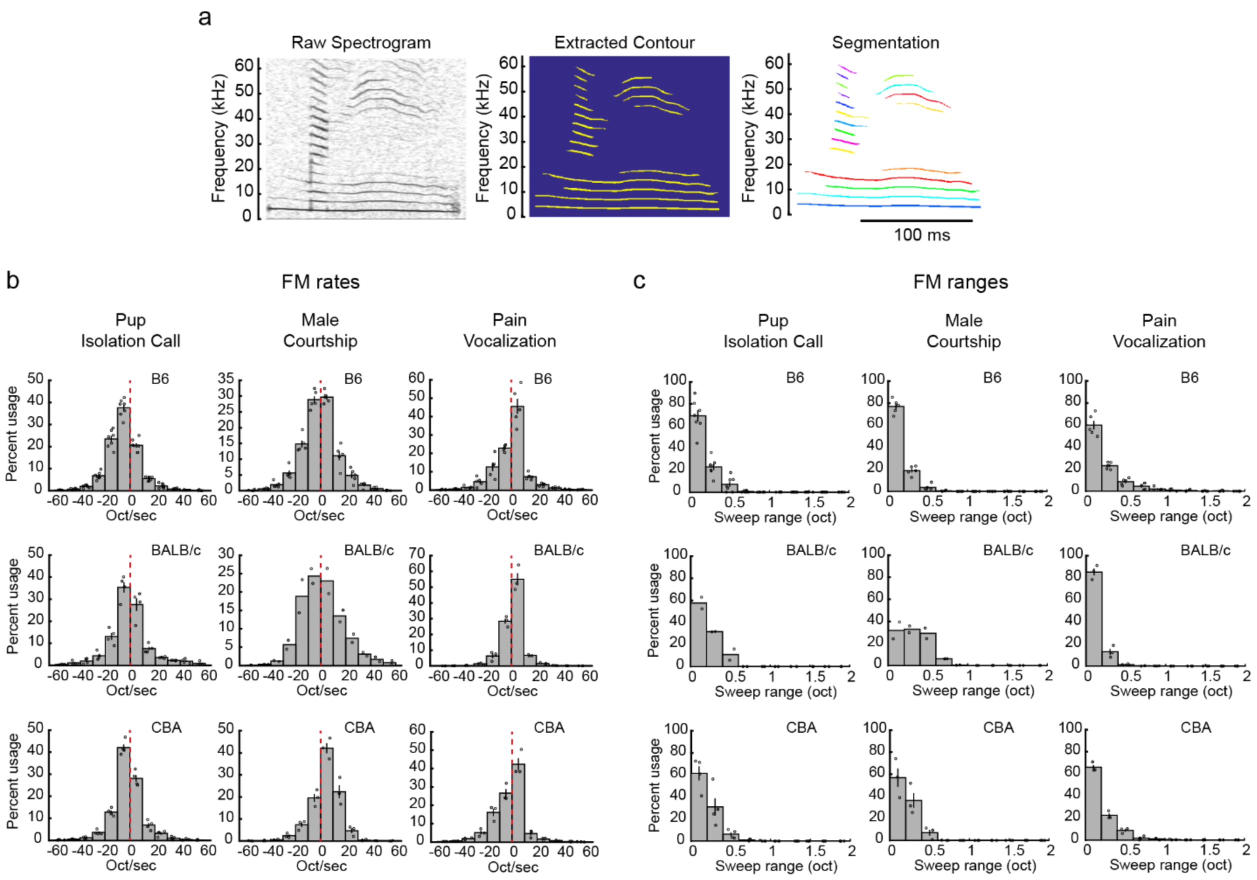

Supplementary Figure 1. Quantification of FM rates and ranges in mouse vocalizations. (a) Left, raw spectrogram of a representative pain vocalization syllable which contains multiple frequency components. Middle, extracted contour of the syllable. Right, automatically detected continuous frequency components individually color-coded to show the successful segregation. FM ranges are calculated from the entire trajectory of individual continuous components, and individual components are further segmented into 5-ms fragments for the calculation of FM rates. **(b)** Histograms showing the usage probability of FM rates for three vocalization categories in C57BL/6J (top), BALB/c (middle), and CBA (bottom) strains, overlaid with individual data points (B6: n = 7, 5, 5; BALB: n = 4, 2, 3; CBA: n = 4, 3, 3 mice for pup, male, and pain vocalizations. Mice with less than 100 vocalization contour 5-ms fragments were excluded). **(c)** Histograms showing the usage probability of FM ranges for three vocalization categories in three strains overlaid with individual data points (B6: n = 7, 5, 5; BALB: n = 2, 2, 3; CBA: n = 4, 3, 3 mice for pup, male, and pain vocalizations. Mice with less than 100 vocalization contour segments were excluded). Results are mean ± SEM.

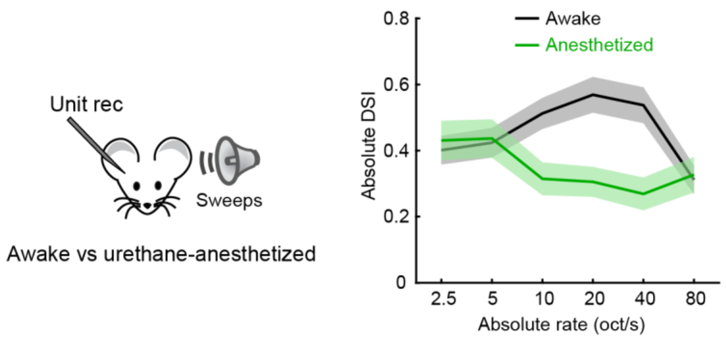

Supplementary Figure 2. Enhanced direction selectivity in the awake state compared to the anesthetized state. Left, schematic of single-unit recordings in awake and urethane-anesthetized mice. Right, average (solid line) and SEM (shading) of absolute DSI at each FM rate in awake (black; n = 8 mice, 80 regular-spiking units) and anesthetized (green; n = 5 mice, 45 regular-spiking units) states. In contrast to a previous study[1], we observed direction selectivity across all FM rates under urethane anesthesia. Nevertheless, we observed an enhancement of DSI in the awake state at middle-range FM rates.

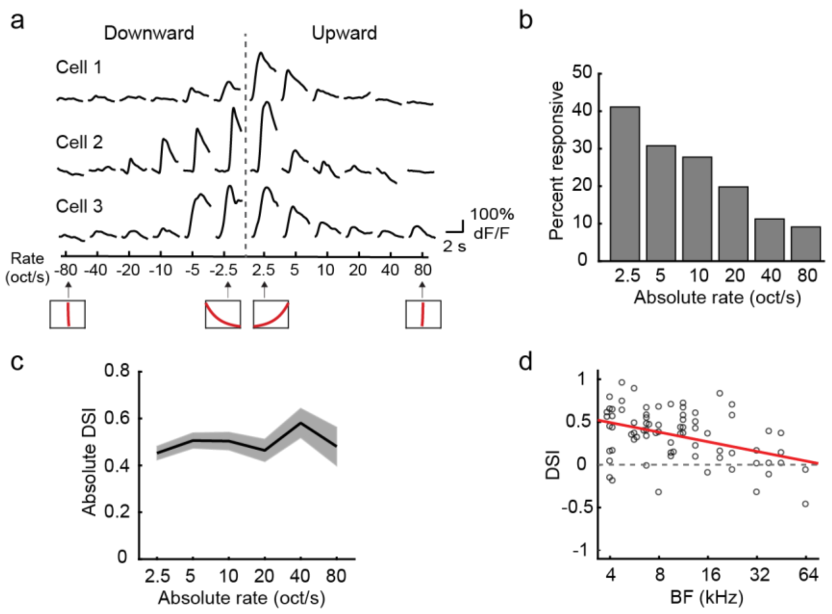

Supplementary Figure 3. VGAT-positive inhibitory neurons show direction selective properties similar to pyramidal cells. (a) FM sweep tuning of three representative L2/3 VGAT cells measured with two-photon calcium imaging. Traces are average responses (five trials) for each FM rate. Insets at the bottom show the schematics of frequency versus time representations. **(b)** Fraction of responsive cells at six absolute FM rates. (n = 8 mice, 328 cells). **(c)** Average (solid line) and SEM (shading) of absolute DSI at each FM rate (n = 8 mice, 100 sweep-responsive cells). **(d)** DSI of VGAT cells is dependent on their BF (n = 8 mice, 74 cells responsive to both sweeps and pure tones. R = -0.416, p = 0.0002, two-sided t-test). Red line, regression curve.

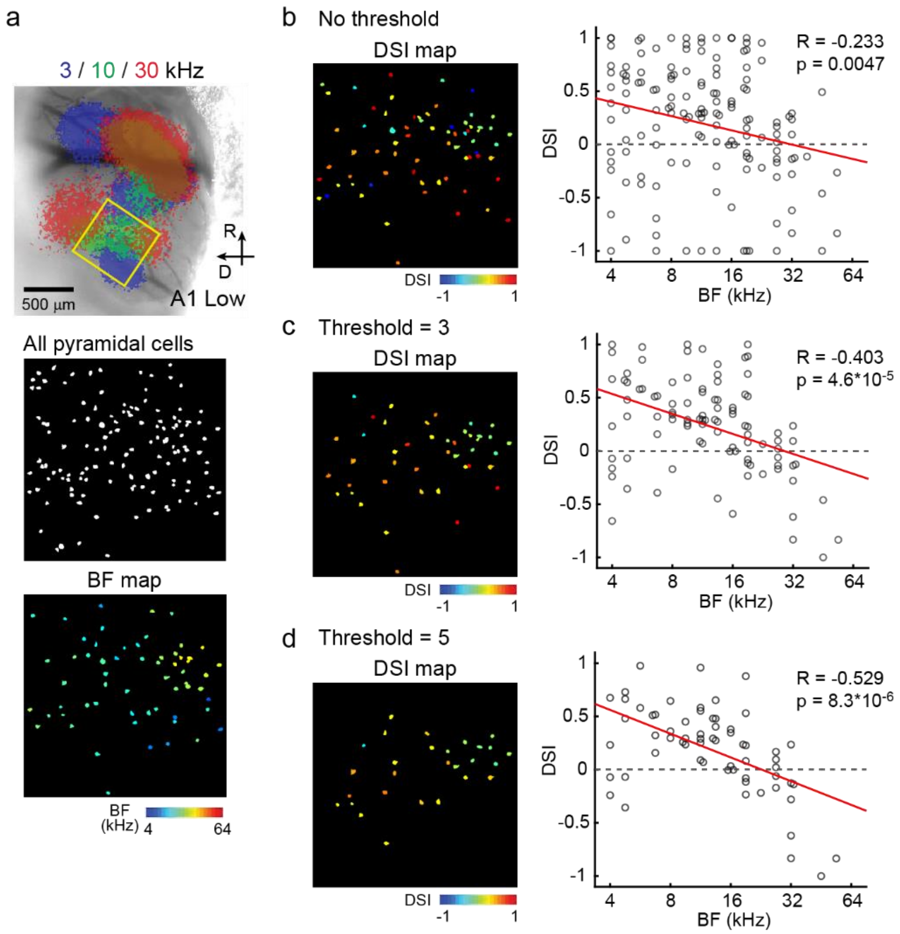

Supplementary Figure 4. Local heterogeneity depends on thresholds for data inclusion. (a) Top, intrinsic signal image superimposed on cortical vasculature imaged through a glass window. Yellow square represents the two-photon imaging fields of view. A1 tonotopy was reproducibly observed in all eight mice. Bottom, maps showing the location of imaged pyramidal cells and their BF for pure tones. The maps are the same as the ones shown for A1 Low area in Figure 1g. **(b)** Re-analysis of Fig. 1g and 1h without applying any data inclusion criterion for number of responsive sounds (n = 8 mice, 146 cells, p = 0.0047, two-sided t-test). Red line, regression curve. **(c)** The same data as Fig. 1g and 1h, which includes only cells that are responsive to at least three sounds (n = 96 cells, $p = 4.6 \times 10^{-5}$, two-sided t-test). This improves the exclusion of cells with false-positive responses that arise from random trial variability. **(d)** Re-analysis data of Fig. 1g and 1h, including only cells that are responsive to at least five sounds (n = 63 cells, $p = 8.3 \times 10^{-6}$, two-sided t-test). Note that fitting improves as higher thresholds are applied for data selection, indicating that cells with robust sweep responses more strictly follow the global DSI-BF relationship.

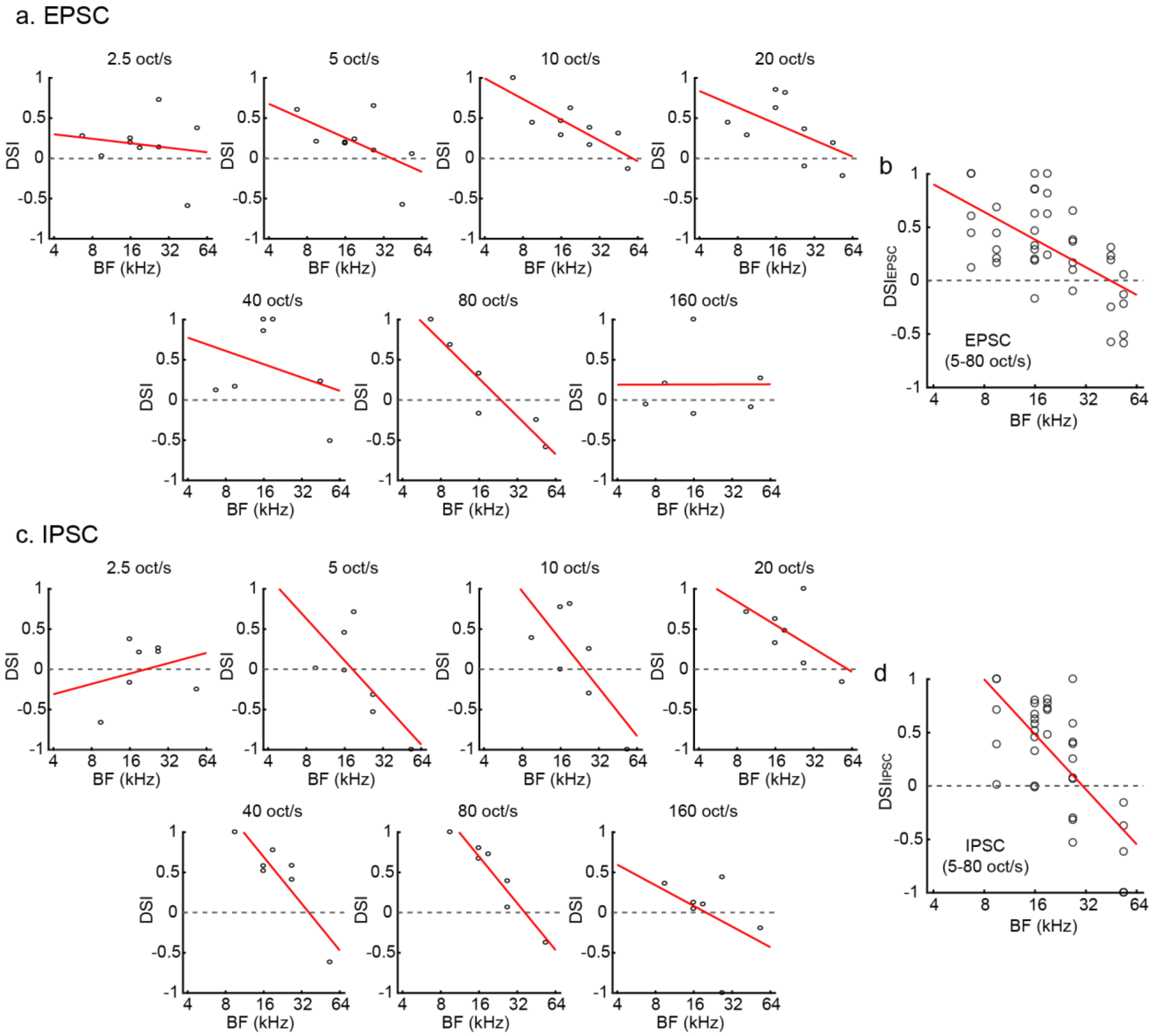

Supplementary Figure 5. DSI of postsynaptic currents have a strong dependence on the BF of the same neurons. (a) BF dependence of DSI_{EPSC} shown separately for individual absolute FM rates (n = 5 mice, 9 cells). Red lines, regression curves. **(b)** BF dependence of DSI_{EPSC} combining all data points for absolute FM rates between 5-80 oct/s (n = 40 cell-rate pairs, R = -0.595, $p = 5 \times 10^{-5}$, two-sided t-test). **(c)** BF dependence of DSI_{IPSC} shown separately for individual absolute FM rates (n = 4 mice, 7 cells). **(d)** BF dependence of DSI_{IPSC} combining all data points for absolute FM rates between 5-80 oct/s (n = 35 cell-rate pairs, R = -0.697, $p = 3 \times 10^{-6}$, two-sided t-test).

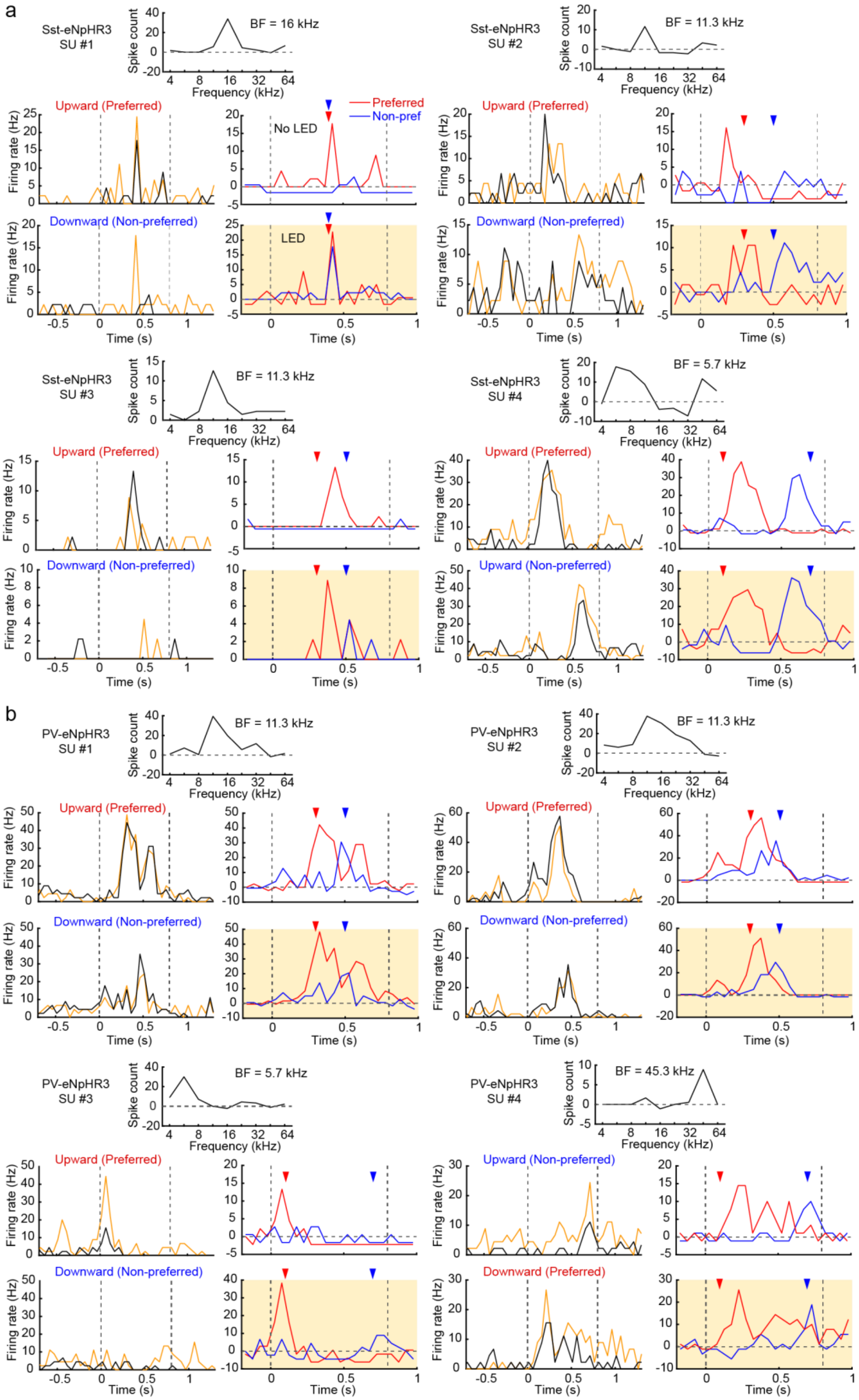
a
Sst-eNpHR3
SU #1
BF = 16 kHz
Spike count
Frequency (kHz)
Upward (Preferred)
Preferred
Non-pref
No LED
Downward (Non-preferred)
LED
Firing rate (Hz)
Time (s)
Sst-eNpHR3
SU #2
BF = 11.3 kHz
Upward (Preferred)
Downward (Non-preferred)
Sst-eNpHR3
SU #3
BF = 11.3 kHz
Upward (Preferred)
Downward (Non-preferred)
Sst-eNpHR3
SU #4
BF = 5.7 kHz
Upward (Preferred)
Upward (Non-preferred)
b
PV-eNpHR3
SU #1
BF = 11.3 kHz
Upward (Preferred)
Downward (Non-preferred)
PV-eNpHR3
SU #2
BF = 11.3 kHz
Upward (Preferred)
Downward (Non-preferred)
PV-eNpHR3
SU #3
BF = 5.7 kHz
Upward (Preferred)
Downward (Non-preferred)
PV-eNpHR3
SU #4
BF = 45.3 kHz
Upward (Non-preferred)
Downward (Preferred)

Supplementary Figure 6. Additional representative single-units for photoinactivation of inhibitory neurons. (a) FM sweep responses of four representative regular-spiking single-units at 5 oct/s with (amber shades and traces) and without (black traces) SOM cell photoinactivation. Top, pure tone tuning properties at 70 dB SPL. Left, peristimulus time histogram (PSTH) of responses to preferred and non-preferred FM directions. Right, PSTHs shown separately for control and photostimulation trials after subtracting the baseline firing rate just before sounds. Arrowheads show the timing of FM sweeps crossing the best frequency (BF) of the recorded units for preferred (red) and non-preferred (blue) directions. SU #1 is the same unit as Fig. 3b and 3c. **(b)** FM sweep responses of four representative regular-spiking single-units with and without PV cell photoinactivation. SU #1 is the same unit as Fig. 3e and 3f.

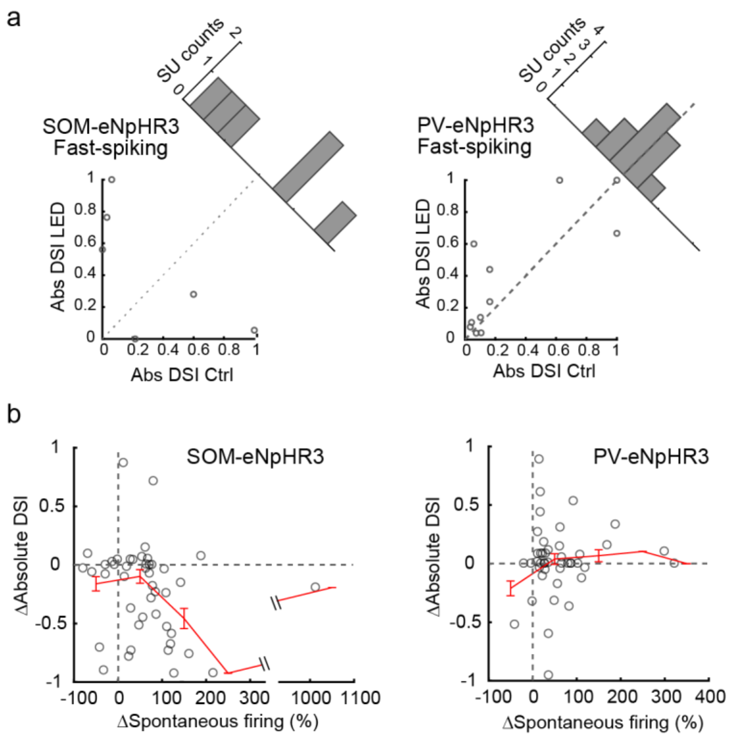

Supplementary Figure 7. Additional data for optogenetic inactivation of inhibitory neurons. (a) Left, scatter plot showing absolute DSI of fast-spiking units during control and SOM cell photoinactivation trials. Gray dots show all sweep-responsive single-units (n = 4 mice, 8 fast-spiking single-units). The oblique histogram illustrates the changes in absolute DSI with LED ($p = 0.693$, two-sided t-test). Right, the same plots for PV cell photoinactivation. n = 4 mice, 13 fast-spiking single-units. ($p = 0.238$). Dotted lines, unity lines. **(b)** Left, scatter plot showing absolute DSI change against spontaneous firing rate change triggered by SOM cell photoinactivation in individual regular-spiking units (n = 45 regular-spiking units). Red line and error bars represent mean ± SEM for each 100-percent bin of spontaneous firing change. Units with spontaneous firing rate less than 0.25 Hz were excluded. Right, the same plot for PV cell photoinactivation (n = 44 regular-spiking units).

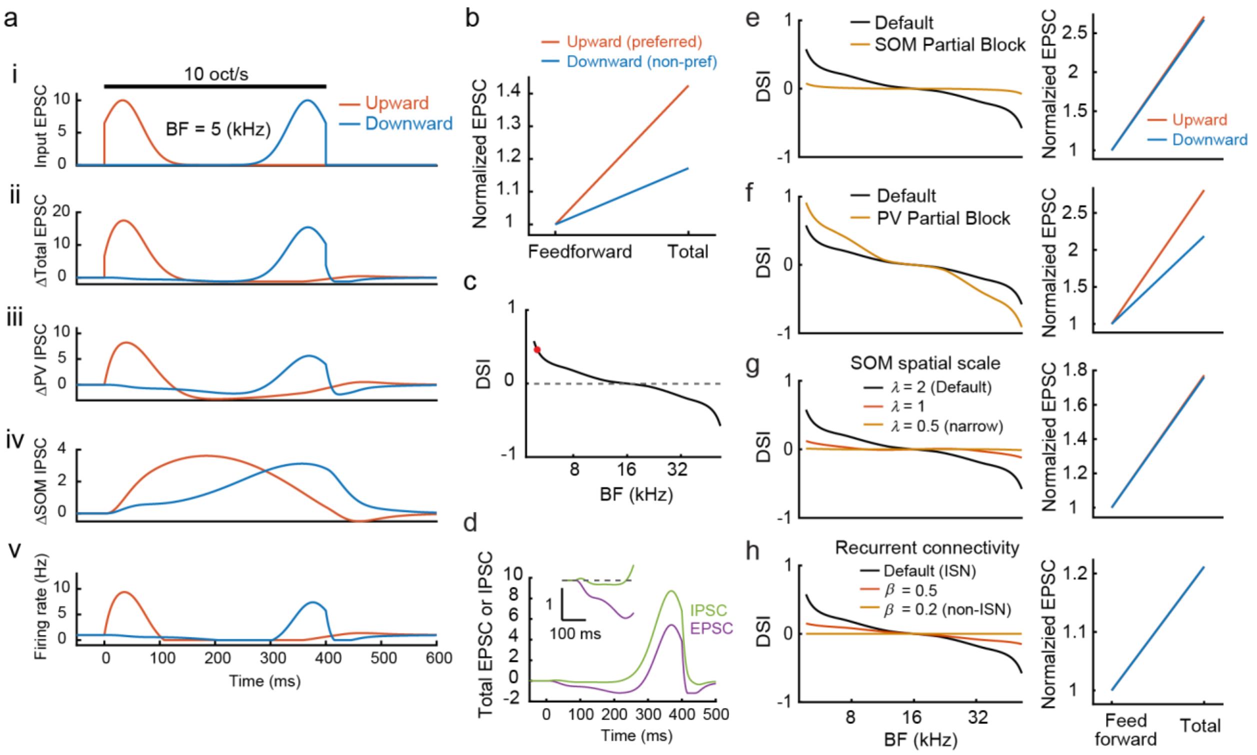

Supplementary Figure 8. Simulation results using 10 oct/s FM sweeps. (a) Time courses of feedforward EPSC (i), change in total EPSC (ii), change in IPSC from PV cells (iii), change in IPSC from SOM cells (iv), and firing rate (v) of a pyramidal cell with 5 kHz BF. Dark line represents FM sweep stimulus at ±10 oct/s. **(b)** Summary plot showing a differential amplification of excitatory postsynaptic charges between upward and downward directions. Amplitudes are normalized to that of feedforward EPSC. **(c)** DSI plotted against BF at ±10 oct/s FM sweeps. Data point with BF = 5 kHz is shown as a red dot. **(d)** Time courses of total EPSC (purple) and total IPSC (green) triggered by a downward 10 oct/s FM sweep in a pyramidal cell with 5 kHz BF. Inset shows a magnified view of the leading network suppression, where both EPSC and IPSC are suppressed below baseline level. **(e)** Left, DSI plotted against BF at ±10 oct/s FM rates in the control (black) and SOM cell partial inactivation (yellow) conditions. Right, amplification of excitatory postsynaptic charges in response to upward and downward direction FM sweeps. **(f)** Results for PV cell partial inactivation. **(g)** Results for reduced spatial scales of SOM cell connectivity. **(h)** Results for non-ISN models with reduced recurrent excitation strengths.

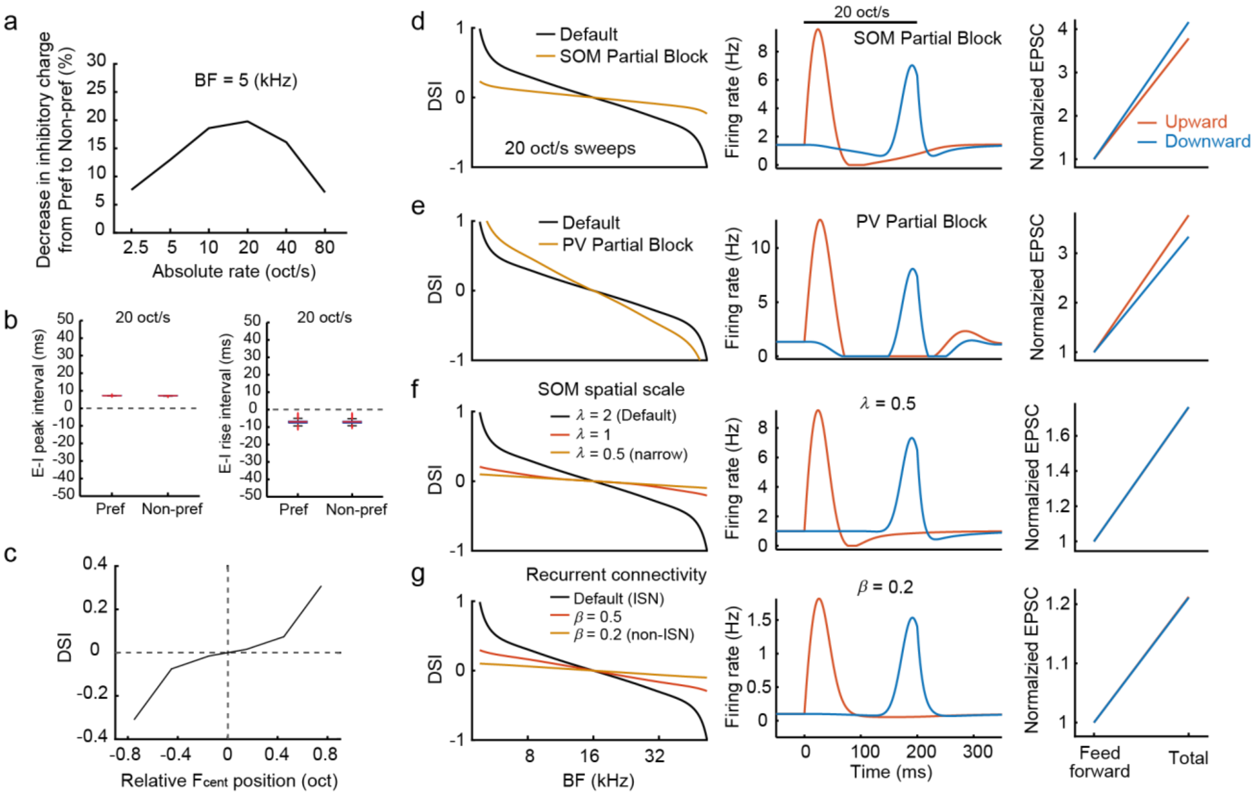

Supplementary Figure 9. Additional modeling data. **(a)** Suppression of FM sweep-triggered inhibitory charge triggered by non-preferred direction compared to that by preferred direction, plotted against absolute FM rates. **(b)** Left, time by which EPSC peak precedes IPSC peak is not different between preferred (7.20 ms) and non-preferred (7.17 ms) direction. Right, time by which EPSC onset precedes IPSC onset is not different between preferred (-7.06 ms) and non-preferred (-7.12 ms) direction. Box and whisker plots represent data points from 401 locations along the tonotopic axis, with median and 25th and 75th percentiles as box edges and 1.5 × interquartile range as whiskers. Red crosses show outliers. **(c)** Averaged DSI plotted for each bin of relative F_{cent} position, showing the reversal of DSI sign. The plot lacks the data points that return to DSI of zero, since our modelled cells have fixed narrow bandwidth of excitatory input and thus do not respond to FM sweeps outside this range. **(d-g)** Data from the network model with inherited direction selectivity in the feedforward input. **(d)** Left, DSI plotted against BF at ±20 oct/s FM rates in the control (black) and SOM cell partial inactivation (yellow) conditions. Middle, firing rate of a pyramidal cell (5 kHz BF) during SOM cell partial inactivation. Right, amplification of excitatory postsynaptic charges in response to upward and downward FM

directions. **(e)** Results for PV cell partial inactivation. **(f)** Results for reduced spatial scales of SOM cell connectivity. **(g)** Results for non-ISN models with reduced recurrent excitation strengths. There is residual direction selectivity even after SOM cell inactivation, reduced spatial scales of SOM cell connectivity, or reduced recurrent excitation strengths, showing the inheritance of direction selectivity from the input.

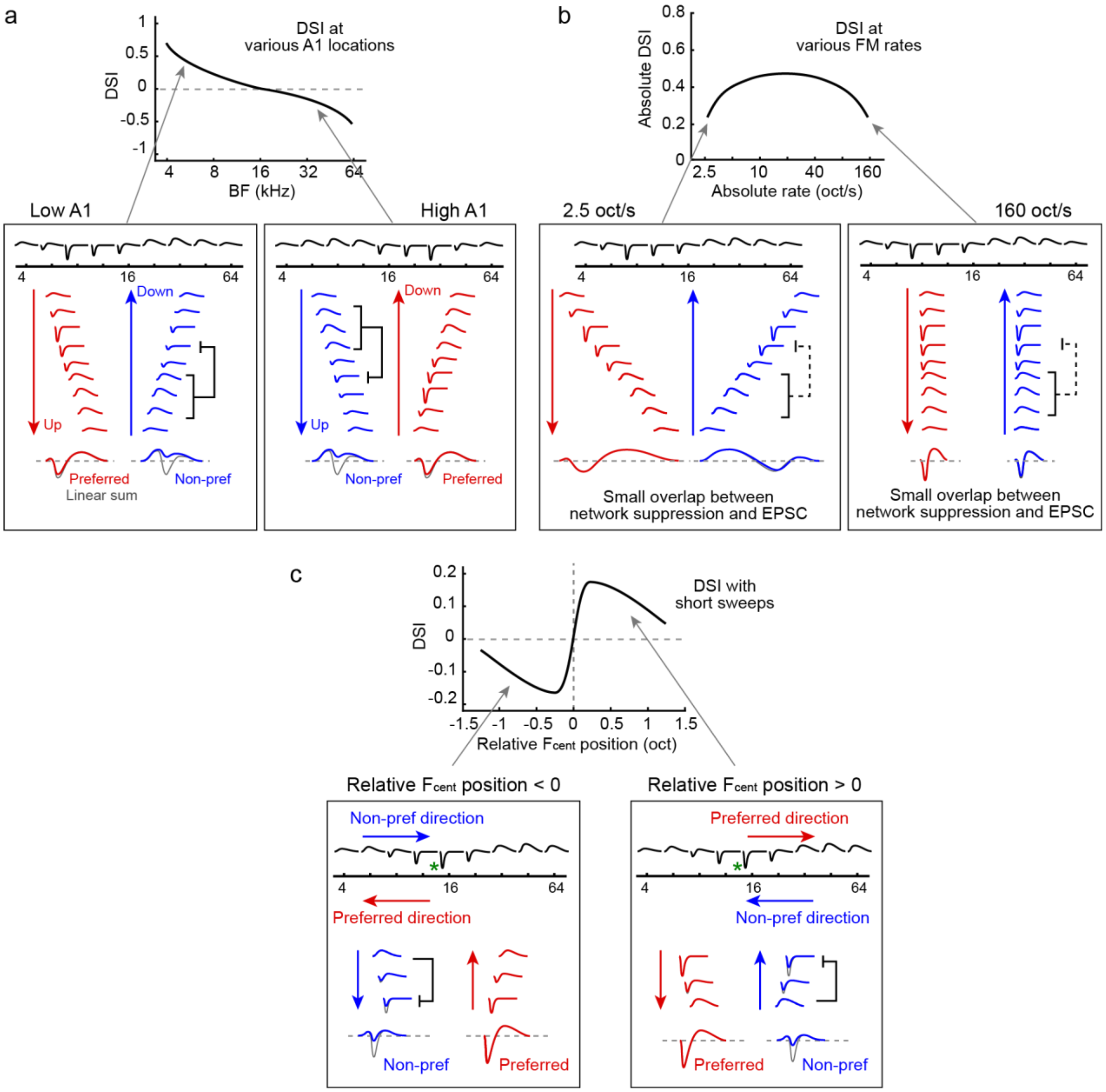

Supplementary Figure 10. Schematic drawings for the generation of direction selectivity in different experimental conditions. (a) Schematics illustrating the generation of upward preference in a low-BF neuron and downward preference in a high-BF neuron. Top, schematic showing the BF dependence of DSI. Bottom left, pure tone responses of a low-BF neuron staggered to account for the frequency movements during FM sweeps. In downward direction, network suppression overlaps with fast EPSC and attenuates it, resulting in an upward preference. Bottom right, the same schematic for a high-BF neuron. Network suppression overlaps with fast EPSC in upward direction, resulting in a downward preference. **(b)**

Schematics illustrating the limited direction selectivity at extremely low (left) and high (right) FM rates. Top, schematic showing the FM rate dependence of DSI. Bottom left, pure tone responses staggered to account for the slow FM sweeps. Due to big temporal offsets between frequencies, network suppression does not overlap with fast EPSC, thus failing to attenuate EPSCs. Bottom right, pure tone responses staggered to account for the fast FM sweeps. Due to small temporal offsets between frequencies, fast EPSC is evoked before network suppression regardless of the directions, thus failing to attenuate EPSCs. **(c)** Schematics illustrating the reversal of direction selectivity depending on the FM frequency range. Bottom, pure tone responses staggered to account for the FM sweeps. In the FM direction toward BF (blue), network suppression overlaps with fast EPSC and attenuates it. In contrast, in the FM direction away from BF (red), fast EPSC precedes network suppression, and thus there is no attenuation of EPSCs.

Supplementary Table 1. Effective interaction strength from population $\boldsymbol{\beta}$ (columns) to $\boldsymbol{\alpha}$ (rows).

$W_{\alpha\beta}$	E	PV	SOM
E	1.2	-1.5	-1.5
PV	1.2	-1.2	-1.2
SOM	2.5	0	0

Supplementary Table 2. Spatial projection width from population $\boldsymbol{\beta}$ (columns) to $\boldsymbol{\alpha}$ (rows).

$\lambda_{\alpha\beta}$	E	PV	SOM
E	0.5	0.5	2
PV	0.5	0.5	2
SOM	2	-	-

Supplementary Table 3. Default parameter values.

Parameter	Default Value	Description
$[\tau_E^m, \tau_P^m, \tau_S^m]$	[10, 10, 10] (ms)	Rate time constant
$[\tau_E^r, \tau_P^r, \tau_S^r]$	[0, 0, 100] (ms)	Temporal filter time constant
$[\mathrm{Amp_E}, \mathrm{Amp_P}, \mathrm{Amp_S}]$	[10, 7, 0] (Hz)	Feedforward amplitude
$[\sigma_E, \sigma_P, \sigma_S]$	[0.5, 0.5, -] (1/oct^2)	Feedforward stimulus width
r_E^0	1 (Hz)	Baseline firing rate of E population

Supplementary Text.

Proof of lack of direction selectivity in a system with linear neuronal transfer function.

Assume that the change of the firing rate from baseline can be described by the following linear system

$$\mathbf{T}\frac{d\Delta\mathbf{r}}{dt} = -\Delta\mathbf{r} + \mathbf{W}\Delta\mathbf{r} + \mathbf{b}(t), \tag{10}$$

and define the DSI be

$$\mathrm{DSI} = \frac{\int_0^\infty \Delta r_E^{up}dt - \int_0^\infty \Delta r_E^{down}dt}{\int_0^\infty \Delta r_E^{up}dt + \int_0^\infty \Delta r_E^{down}dt} = \frac{\Delta R_E^{up} - \Delta R_E^{down}}{\Delta R_E^{up} + \Delta R_E^{down}}. \tag{11}$$

Integrating the equation above yields

$$\int_0^\infty \mathbf{T}\frac{d\Delta\mathbf{r}}{dt}dt = \int_0^\infty -\Delta\mathbf{r} + \int_0^\infty \mathbf{W}\Delta\mathbf{r}dt + \int_0^\infty \mathbf{b}(t)dt. \tag{12}$$

Using the Fundamental Theory of Calculus, the first term of the left-hand side is

$$\int_0^\infty \mathbf{T}\frac{d\Delta\mathbf{r}}{dt}dt = \mathbf{T}\left[\lim_{t\to\infty}\Delta\mathbf{r}(t) - \Delta\mathbf{r}(0)\right] = 0. \tag{13}$$

Simplifying the remaining terms of the equation yields

$$0 = (\mathbf{I} - \mathbf{W})\Delta\mathbf{R} + \mathbf{B}, \tag{14}$$

$$\Rightarrow \Delta\mathbf{R} = (\mathbf{I} - \mathbf{W})^{-1}\mathbf{B}. \tag{15}$$

Since $\mathbf{B}^{up} = \mathbf{B}^{down}$ (even though the time courses $\mathbf{b}(t)^{up} \neq \mathbf{b}(t)^{down}$), we find that $\Delta R_E^{up} = \Delta R_E^{down}$, and thus DSI is equal to 0.

Examining the change in baseline with the non-linear model.

Since DSI requires only the change in baseline firing rate, the parameters μ_α superfluous to this study. To see this, let r_α^0 denote the steady state firing rates of the system when $b_\alpha(x,t) = 0$ and denote the change from baseline as ΔA_α. Substituting $\Delta A_\alpha + r_\alpha^0$ into our ODE for ΔA_α yields

$$\tau_\alpha^m \frac{d(\Delta A_\alpha + r_\alpha^0)}{dt} = -(\Delta A_\alpha + r_\alpha^0) + \sum_\beta \int K_{\alpha\beta}(x,y) F\left(\Delta r_\beta(y) + r_\alpha^0\right) dy + b_\alpha(x,t) + \mu_\alpha. \quad (16)$$

This equation can then be simplified and rewritten as

$$\tau_\alpha^m \frac{d\Delta A_\alpha}{dt} = -\Delta A_\alpha + \sum_\beta \int K_{\alpha\beta}(x,y) \hat{F}_\beta\left(\Delta r_\beta(y)\right) dy + b_\alpha(x,t) + \left[\sum_\beta W_{\alpha\beta} r_\beta^0 + \mu_\alpha - r_\alpha^0\right], \quad (17)$$

where

$$\hat{F}_\beta(x) = \begin{cases} x & \text{if } x > -r_\beta^0 \\ -r_\beta^0 & \text{otherwise} \end{cases}. \quad (18)$$

Since r_α^0 is the steady state solution, the terms in the bracket sum up to zero, eliminating the parameters μ_α from appearing. Furthermore, the baseline firing rates only appear in the adjusted thresholding function $\hat{F}_\beta(x)$. We show that for DSI to be non-zero, this non-linear threshold must come into effect for at least the E populations. As a result, for simplicity we assume that the PV and SOM equations operate solely in the linear regime, resulting in r_P^0 and r_S^0 also being superfluous parameters that need not be specified.

Discussion on the contribution of intensity ramp preference

In the mouse auditory system, hearing sensitivity is the highest around 10-30 kHz and decreases towards the edges of the hearing range. Therefore, FM sweeps with a constant sound level will nevertheless provide an input that changes not only on the sound frequency, but also on the intensity that the system perceives. A previous study found that mouse auditory cortex neurons (including A1 and other areas) preferentially respond to intensity ramp-up, as opposed to ramp-down, sounds with fixed spectral content[2]. This raises a question whether the observed FM direction selectivity in A1 neurons is due to the perceived ramps in the sound intensity. We believe that our main findings are not explained by this mechanism for the following reasons:

Although the circuit mechanisms underlying the intensity ramp-up preference are unknown, we can think of two alternative scenarios: 1) intensity ramp-up preference is calculated within each neuron, such that ramp-up of the synaptic input onto each neuron is critical for this computation, or 2) intensity ramp-up preference is calculated in the whole network, such that ramp-up of the overall inputs to the auditory cortex is critical for this computation.

Scenario 1) is obviously against our main findings on 4-octave FM sweeps data. A neuron in low-frequency A1 (e.g. BF = 4 kHz) would experience a ramp-down of synaptic inputs for upward FM sweeps and a ramp-up of synaptic inputs for downward FM sweeps. Therefore, this scenario would predict a downward FM sweep preference in low frequency A1 neurons, which is completely the opposite of our findings in Fig. 1h.

Scenario 2), in contrast, could contribute to the results in Fig. 1h. However, this scenario in turn fails to explain our results on spectrally-restricted FM sweeps (Fig. 6), since this scenario predicts fixed DSI of individual neurons regardless of the FM frequency range. For example,

upward FM sweeps of 4→8 kHz and 8→16 kHz both cause an intensity ramp-up of overall inputs to the auditory cortex, thus there should not be a difference in DSI between these two FM stimuli in individual neurons. What we demonstrated in Fig. 6b and 6e is clearly inconsistent with this prediction—we showed that DSI of individual neurons is not a fixed value but rather depends on the relationship between the neuron's BF and the FM frequency range. For example, a neuron with 8 kHz BF would show a downward-preference for 4-8 kHz FM range and an upward-preference for 8-16 kHz FM range.

Together, in either scenario, intensity ramp-up preference fails to account for our results on FM sweep direction selectivity. However, we do not exclude the possibility that this mechanism, together with other direction selectivity mechanisms, such as delay-and-compare between ON and OFF responses, combination-sensitive supralinear summation, and inheritance from upstream structures, contributes to the local heterogeneity in DSI that are not captured by the global trend that our model explains.

1. Zhang, L. I., Tan, A. Y. Y., Schreiner, C. E. & Merzenich, M. M. Topography and synaptic shaping of direction selectivity in primary auditory cortex. *Nature* **424**, 201–205 (2003).

2. Deneux, T., Kempf, A., Daret, A., Ponsot, E. & Bathellier, B. Temporal asymmetries in auditory coding and perception reflect multi-layered nonlinearities. *Nat. Commun.* **7**, 12682 (2016).
