# Supplementary material for: Recurrent network dynamics shape direction selectivity in primary auditory cortex
Source: Nat Commun. 2021 Jan 12;12:314. doi: 10.1038/s41467-020-20590-6 (PMC7804939; doi:10.1038/s41467-020-20590-6)
Supplement: Supplementary file 3 — Description of Additional Supplementary Files [file 41467_2020_20590_MOESM3_ESM.pdf]

Description of Additional Supplementary Files

File Name: Supplementary Data 1
Description: **Summary of statistics used in all figures**. Number of data points, statistical test, p value, as well as inclusion and exclusion criteria are listed for each experiment in all figures.

File Name: Supplementary Movie 1
Description: **Simulation of an upward FM sweep as a travelling Gaussian pulse**. Left, time-lapse movie showing changes in firing rate (blue, in Hz), IPSC from PV cells (red, arbitrary unit), IPSC from SOM cells (yellow, arbitrary unit), and feedforward EPSC from thalamus (dashed gray, arbitrary unit) of pyramidal cells along the A1 tonotopic axis over time. 10 oct/s upward FM sweep starts at 0 ms. Middle, firing rate of a pyramidal cell with 5 kHz BF. Moving red dot represents the time point corresponding to the time-lapse movie. Right, firing rate of a pyramidal cell with 60 kHz BF.
