# Supplementary material for: Recurrent network dynamics shape direction selectivity in primary auditory cortex
Source: Nat Commun. 2021 Jan 12;12:314. doi: 10.1038/s41467-020-20590-6 (PMC7804939; doi:10.1038/s41467-020-20590-6)
Supplement: Supplementary file 6 — Reporting Summary [file 41467_2020_20590_MOESM6_ESM.pdf]

nature research

Corresponding author(s): Hiroyuki Kato

Last updated by author(s): Nov 16, 2020

Reporting Summary

Nature Research wishes to improve the reproducibility of the work that we publish. This form provides structure for consistency and transparency in reporting. For further information on Nature Research policies, see Authors & Referees and the Editorial Policy Checklist .

Statistics

For all statistical analyses, confirm that the following items are present in the figure legend, table legend, main text, or Methods section.

n/a	Confirmed	
☐	☒	The exact sample size (*n*) for each experimental group/condition, given as a discrete number and unit of measurement
☐	☒	A statement on whether measurements were taken from distinct samples or whether the same sample was measured repeatedly
☐	☒	The statistical test(s) used AND whether they are one- or two-sided<br>*Only common tests should be described solely by name; describe more complex techniques in the Methods section.*
☐	☒	A description of all covariates tested
☐	☒	A description of any assumptions or corrections, such as tests of normality and adjustment for multiple comparisons
☐	☒	A full description of the statistical parameters including central tendency (e.g. means) or other basic estimates (e.g. regression coefficient) AND variation (e.g. standard deviation) or associated estimates of uncertainty (e.g. confidence intervals)
☐	☒	For null hypothesis testing, the test statistic (e.g. *F*, *t*, *r*) with confidence intervals, effect sizes, degrees of freedom and *P* value noted<br>*Give P values as exact values whenever suitable.*
☒	☐	For Bayesian analysis, information on the choice of priors and Markov chain Monte Carlo settings
☒	☐	For hierarchical and complex designs, identification of the appropriate level for tests and full reporting of outcomes
☐	☒	Estimates of effect sizes (e.g. Cohen's *d*, Pearson's *r*), indicating how they were calculated

Our web collection on statistics for biologists contains articles on many of the points above.

Software and code

Policy information about availability of computer code

Data collection	Matlab (2013a,2016b)<br>ScanImage 5.2<br>Bpod 1.58<br>Wavesurfer 0.945<br>Open Ephys 4.5<br>pClamp 10.7<br>Avisoft-RECORDER 2.97
Data analysis	Matlab (2013a,2016b, 2019a, 2020b)<br>Kilosort 1.0<br>Phy2<br>Fiji (Win 64-bit)<br>Suite2P (2017)<br>The code for all simulations can be found on GitHub (https://github.com/gregoryhandy).

For manuscripts utilizing custom algorithms or software that are central to the research but not yet described in published literature, software must be made available to editors/reviewers. We strongly encourage code deposition in a community repository (e.g. GitHub). See the Nature Research guidelines for submitting code & software for further information.

Data

Policy information about availability of data

All manuscripts must include a data availability statement. This statement should provide the following information, where applicable:

- Accession codes, unique identifiers, or web links for publicly available datasets
- A list of figures that have associated raw data
- A description of any restrictions on data availability

Source Data for all figures are provided as a supplementary data file. Other data that support the findings of this study will be made available from the corresponding author upon reasonable request.

Field-specific reporting

Please select the one below that is the best fit for your research. If you are not sure, read the appropriate sections before making your selection.

[x] Life sciences [ ] Behavioural & social sciences [ ] Ecological, evolutionary & environmental sciences

For a reference copy of the document with all sections, see nature.com/documents/nr-reporting-summary-flat.pdf

Life sciences study design

All studies must disclose on these points even when the disclosure is negative.

Sample size	No methods were used to predetermine sample sizes, but our samples sizes reflect those in previous publications. Specifically, 6-8 mice were used in each two-photon calcium imaging experiment, which results in 500-1000 recorded cells (Komiyama et al., Nature 464, 1182; Kato et al., Neuron 88, 1027). 4 mice were used in each unit recording experiment, which results in around 100 recorded single units (Adesnik et al., Nature 490, 226; Reinhold et al., Nat Neurosci 18, 1789). 7-9 cells in 4-5 mice were recorded in in vivo whole-cell recording experiment (Zhou et al., Nat Neurosci 17, 841; Wehr and Zador, Neuron 47, 437 ; Kato et al., Neuron 95, 412). The exact number of animals are given in the main text and also in figure legends.
Data exclusions	In calcium imaging, pyramidal cells whose nuclei were filled with GCaMP were excluded from analyses. In whole-cell recordings, experiments for cells with unstable series resistance and cells without clear sound-evoked excitatory currents were terminated and thus excluded. During spike sorting, single units with less than 1000 spikes were excluded from the study. Cells and units that did not exhibit statistically significant responses to any auditory stimuli are reported, but their tuning was not analyzed. These exclusion criteria are standard in the field and were pre-established before experiments.<br>In mouse vocalization statistics, mice with <100 vocal contour segments were excluded from the calculation of histogram. Units that showed <0.25 Hz baseline firing were excluded from the calculation of optogenetics-dependent change in spontaneous firing rate. These exclusion criteria were not pre-established.
Replication	Results described in the paper were reproduced in multiple rounds of experimentations. Data were always acquired from multiple mice and multiple litters, and two-photon calcium imaging data were collected by two independent researchers. No issues were identified in reproducing any of the reported findings, as shown in the scatter plots or the Source Data. The exact number of repetitions are indicated in text, figure legends, and Supplementary Table 4.
Randomization	The study did not entail separating animals into different experimental groups (e.g. control vs. experimental). Thus, there was no randomization. Optogenetic manipulation was performed in interleaved trials.
Blinding	A critical step in which bias can potentially occur is in spike sorting, in which individual units are included or excluded by the number of spikes they exhibit, spike waveform separation from noise, etc. We performed spike sorting blind to any stimulus- or location-related information. Thus, a unit's properties were not known at the time of spike sorting. For the quantification of FM sweeps-triggered fast synaptic currents, onsets and offsets of events were determined blind to the directions of FM sweeps with the help of a custom GUI. For comparison between optogenetic and control conditions, blinding during data collection was not necessary to maintain integrity in our data since LED and control trials were always randomly interleaved. For other experiments, blinding was not relevant since there was no group allocation during data collection.

Reporting for specific materials, systems and methods

We require information from authors about some types of materials, experimental systems and methods used in many studies. Here, indicate whether each material, system or method listed is relevant to your study. If you are not sure if a list item applies to your research, read the appropriate section before selecting a response.

Materials & experimental systems

n/a	Involved in the study
☒	☐ Antibodies
☒	☐ Eukaryotic cell lines
☒	☐ Palaeontology
☐	☒ Animals and other organisms
☒	☐ Human research participants
☒	☐ Clinical data

Methods

n/a	Involved in the study
☒	☐ ChIP-seq
☒	☐ Flow cytometry
☒	☐ MRI-based neuroimaging

Animals and other organisms

Policy information about studies involving animals; ARRIVE guidelines recommended for reporting animal research

Laboratory animals	Mice were at least 6 weeks old at the time of experiments. Mice were acquired from Jackson Laboratories (VGAT-Cre, SOM-Cre, PV-Cre, ROSA-LSL-tdTomato, C57BL/6J, CBA/J, and BALB/cJ) or Charles River Laboratories (C57BL/6J, CBA/J, and BALB/cJ). Both female and male animals were used.
Wild animals	Study did not involve wild animals.
Field-collected samples	Study did not involve collections from the field.
Ethics oversight	All procedures were in accordance with the Institutional Animal Care and Use Committee at the University of North Carolina at Chapel Hill and Osaka University as well as guidelines of the National Institute of Health.

Note that full information on the approval of the study protocol must also be provided in the manuscript.
